# Bio-fertilizers ameliorate drought tolerance and physio-biochemical attributes of *Moringa oleifera* plants under different irrigation scheduling

**DOI:** 10.1186/s12870-026-09333-9

**Published:** 2026-07-09

**Authors:** Rasha M. El-Shazoly, Mohamed A. Youssef, Asmaa M. El-Zohary, Muhammad M. El-Sayed, Muhammad Saqlain Zaheer, Mahmoud M. El-sayed

**Affiliations:** 1 Botany and Microbiology Department, Faculty of Science, New Valley University, New Valley, Al-Kharja 72511 Egypt; 2 Soils and Water Department, Faculty of Agriculture, Al-Azhar University, Assiut, 71524 Egypt; 3 Technology, Engineering and Mathematics School, Assiut, 71511 Egypt; 4 Department of Agricultural Engineering, Khwaja Fareed University of Engineering and Information Technology, Rahim Yar Khan, Pakistan

**Keywords:** Fertilization, PGPR, Soil moisture deficit, Yield components, Enzymatic and—non-enzymatic antioxidants

## Abstract

Increased drought stress is anticipated to impact crop productivity adversely, mainly in Africa and Southeast Asia. Drought-tolerant plant growth-promoting rhizobacteria (PGPR) have emerged as a ray of hope as an effective bio-enhancer and sustainable strategy for agriculture under water-deficit and recovery conditions to reduce reliance on chemical fertilizers and improve soil fertility. Keeping this in view, a field experiment was conducted to check the effectiveness of commercial bio-fertilizers Phosphorine, Microbine, Potassiumag individually, or in combination with organic fertilizer (compost), and natural mineral rock (rock phosphate), as a substituent of chemical fertilizer. Two drip irrigation schedules W_1_ (100%) and W_2_ (60% of field capacity FC) were applied to evaluate growth and biochemical traits of *Moringa oleifera* under varying biofertilizer inputs. The highest values of plant growth under W_1_ were recorded in the plants amended with BioMix1 (Microbine mixed with rock phosphate and compost), plant height (150 cm), number of branch plant^−1^ (19), plant dry weight (203 g). On the other hand, individual application of Potassiumag (Bio_3_) showed the highest of plant growth under W_2_ water scheduling, with plant height (130 cm), number of branch plant^−1^ (17), plant dry weight (137 g). The present work evaluated a broad range of both non-enzymatic antioxidant components including phenolics, proline, ascorbate and carotenoids, in addition to peroxidase (POD), ascorbate peroxidase (APX) and catalase (CAT) enzymes. Microbine with compost and rock phosphate improved *Moringa oleifera* yield and quality at 100% FC, while Potassiumag followed by Phosphorine sustained growth under drought 60%FC.

## Introduction

*Moringa oleifera* (also known as *Moringa pterygosperma* Gaertn) belongs to the Moringaceae family. This species of moringa is native from south Himalaya region [1], *Moringa oleifera* leaves, consumed fresh or processed into powders and teas, are rich in proteins, vitamins, minerals, and antioxidants, with their phenolic content and antioxidant activity [1]. It is considered as very healthy food source and ethnobotany [2]. Local populations in regions with high susceptibility to desertification consider* M. oleifera* as important crop due to its high nutritional value. It is a valuable commercial species in the agro-pharmaceutical industry for its antioxidant properties [1]. As a consequence, it was introduced in several countries of tropical and subtropical areas due to environmental pressure of increased drought (e.g. Africa, South-east Asian regions) [3, 4]. Drought is a serious threat to plant productivity and food security worldwide [5]. Water stress manipulates plant turgor and water potential, possibly leading to negative consequences in plants morphological and physiological traits [6, 7]. Chloroplast structure and photosynthesis are obviously sensitive to abiotic stresses [8]. The increase of reactive oxygen species (ROS) is a primary cell target of most abiotic stresses and therefore, the antioxidant system is also affected. This has been well documented for plants under drought [8–12].

Medicinal and stress tolerant plants are valued for their phytochemical richness and adaptive capacity under harsh environments. Studies on medicinal species such as *Ruta graveolens* emphasize the role of secondary metabolites and antioxidants, which is comparable to the nutraceutical value of *Moringa oleifera* [13]. Plant performance under drought is strongly influenced by soil organic carbon, microbial communities, salinity patterns, and water availability in arid regions [14–16]. Hydrological modifications, including reservoirs and irrigation systems, further regulate plant water dependency and drought exposure [17]. At the physiological level, stress responses involve transcriptional regulation and antioxidant metabolism [18]. Soil nutrient management and organic amendments modify microbial activity and redox processes, indirectly enhancing plant stress tolerance [19, 20]. Although based on microalgae, studies on *Chlorella* demonstrate how light, nitrogen, and hormonal signals regulate protein and antioxidant related metabolism, providing conceptual insight into stress driven metabolic regulation relevant to higher plants [21, 22].

The ROS overproduction stimulates the enzymatic cellular antioxidant system, including CAT, superoxide dismutase (SOD), POD, or APX. The insufficient antioxidant battery induces redox imbalance and ROS damages, such as excessive oxidation of macromolecules as unwanted consequence. The first target of lipid peroxidation may include cell membrane, whose damage may lead to cell death [4, 12].

Several strategies have been explored; however, major efforts have focused on improving water management and enhancing crop drought tolerance. Among these, drip irrigation (trickle irrigation) represents a highly effective approach for optimizing water use efficiency. By delivering water directly to the root zone through emitters or applicators along a supply line, drip irrigation ensures slow and targeted application, typically reducing water consumption compared with conventional methods such as flood irrigation [23]. Since only the potential root zone is irrigated, drip irrigation has been demonstrated to improve crop productivity and quality, reduce energy costs, improve irrigation efficiency, and reduce water loss by deep percolation. On the other hand, strategies of improving crops drought tolerance through traditional plant breeding and genetic engineering have been previously studied [24]. Developing drought-tolerant varieties through genetic engineering has proven to be a complex task due to the multigenic nature of drought stress tolerance. As an alternative approach for sustainable agriculture under water deficit conditions, the application of drought-tolerant plant growth promoting rhizobacteria (PGPR) has gained attention. PGPR have demonstrated remarkable potential in improving the physiological response of plants to water scarcity and enhancing their endurance in drought stress situations [23–25].

Plant Growth-Promoting Rhizobacteria (PGPR) and bio-fertilizers significantly enhance crop productivity (up to 50%) by improving seedling vigor and soil nutrient accessibility [23–28]. Unlike organic fertilizers derived from manures [28], bio-fertilizers are microbial inoculants that increase crop yields through key mechanisms such as atmospheric nitrogen fixation, phosphorus solubilization via organic acids, and the production of growth regulators like indole acetic acid (IAA) [29–31]. Integrating these indigenous microbes with rock phosphate offers a cost-effective and eco-friendly alternative to expensive, energy-intensive chemical fertilizers, which often deplete soil fertility and cause environmental hazards like nitrate leaching [32–35]. Beyond nutrient supply, bio-fertilizers enhance water uptake and act as biocontrol agents, reducing drought susceptibility [36–39].

Recent studies have demonstrated that combining phosphate-solubilizing fungi or bio-fertilizers (e.g., Microbine and Potassiumag) with organic matter significantly improves the growth, yield, and mineral content of *Moringa oleifera* [40–42]. This approach is increasingly critical as the global demand for chemical fertilizers outpaces supply, with projected deficits reaching millions of tons annually [43–46].

Bio-organic fertilizers, prepared from plant residues and beneficial bacteria, not only sustain food production but also mitigate drought stress by influencing metabolic processes like osmotic adjustment and scavenging reactive oxygen species (ROS). While the role of antioxidants (e.g., phenolics, proline, and ascorbate) in plant stress tolerance and human diet is well-recognized, the synergistic mechanisms—specifically antioxidant modulation and nutrient homeostasis—triggered by multi-strain bio-fertilizers under contrasting irrigation regimes remain poorly understood in *Moringa oleifera*.

Therefore, this study was commenced to bridge this knowledge gap by assessing a comprehensive bio-organic strategy designed to maintain *Moringa oleifera* productivity under drought stress. We examined the subsequent research inquiries: Is a synergistic blend of plant growth-promoting rhizobacteria (PGPR), compost, and rock phosphate a viable substitute for chemical fertilizers during drought conditions? And how much do these bio-enhancers activate the plant's biochemical and physiological defenses?

Therefore we hypothesized that: (i) the mixture of PGPR (Microbine, Phosphorine, and Potassiumag) with organic and mineral amendments (MixBio treatments) could potentially maintain growth and yield comparable to chemical fertilization; (ii) these bio-fertilizers elicit adaptive physiological responses that augment drought resilience, chiefly by modulating the enzymatic antioxidant system; and (iii) such sustainable technologies may enhance the nutritional quality of the leaves by increasing protein and ascorbic acid (Vitamin C) accumulation.

## Materials and methods

### Experimental site and plant material

A field experiment was conducted at the Experimental Farm of the Faculty of Agriculture, Al-Azhar University, Assiut, Egypt. The geographical location of the experimental site is 27^°^ 12^−^ 16.67^=^ N latitude and 31° 09^−^ 36.86^=^ E longitude and at 51 m altitudeabove mean sea level. The study aimed to evaluate the effects of individual and combined applications of commercial biofertilizers; Phosphorine, Microbine, and Potassiumag with organic compost and natural rock phosphate on the growth and physiological performance of *Moringa oleifera* Lam. under two soil moisture regimes.

Selection of these specific bio-fertilizers was done taking into consideration the unique microbial functional properties. Microbine was selected because of its nitrogen-fixing and phosphate-solubilizing bacteria, which help to increase the availability of these two nutrients [47]. Phosphorine was selected because of its high solubilization efficiency of insoluble phosphorus, while Potassiumag was selected because of its unique solubilization ability of potassium, which is of great importance in maintaining the osmotic and stomatal regulation under drought stress [48, 49].

Certified *Moringa oleifera* seeds and commercial biofertilizers were obtained from the Agricultural Research Center (ARC), Ministry of Agriculture, Giza, Egypt. Soil physical and chemical properties of the experimental site were analyzed according to standard procedures described by Black et al. [50] and Page et al. [51], and are presented in (Table 1).

**Table 1:** Physical and chemical properties of the experimental soil

Particle size distribution						Texture grade		Bulk density (Mg m^−3^)	E.C (dSm^−1^) soil past	pH Susp.(1:2.5)
Sand %		**Silt %**		**Clay %**						
50.20		30.40		19.40		Sandy loam		1.48	0.869	8.05
C.E.C (cmol_c_ kg^−1^)		CaCO_3_ (g kg^1^)		O.M (%)		Total-N (%)		Ava-N (mg kg^−1^)	Ava-P (mg kg^−1^)	Ava-K (mg kg^−1^)
13.78		11.7		1.38		0.19		69.72	13.58	159.42
Cations (cmol kg^−1^ soil)				Anions (cmol kg^−1^ soil)				Moisture content		Available water (AW)
Ca^++^	Mg^++^	Na^+^	K^+^	CO_3_^−-^	HCO_3_^−^	Cl^−^	SO_4_^−-^	Field capacity (FC)	Welting point (WP)	
0.78	0.64	0.29	0.21	–-	0.57	0.69	0.69	39	19	20

Each value in this table is the mean of 3 replicates

### Fertilization treatments

Seven fertilization treatments were applied, including individual biofertilizers and their combinations with compost and rock phosphate. The detailed description of fertilization treatments is presented in (Table 2). Characteristics and chemical composition of the applied biofertilizers, compost, and rock phosphate are shown in (Table 3). Rock phosphate was obtained from Mangabad Super Phosphate Factory (Assiut, Egypt), while compost was sourced from the ECARU Agricultural Waste Compost Factory, New Minya City, Egypt.

**Table 2:** Treatments code and the combinations fertilization treatments applied per plot

No	Treatment code	Components of treatment
1	CF	Recommended dose of chemical NPK fertilizers at the (240 kg N, 50 kg P_2_O_5_ and 60 kg K_2_O ha^−1^ per cut), added in the form of ammonium nitrate (33.5% N), superphosphate (15.5% P_2_O_5_) and potassium sulphate (48% K_2_O)
2	MixBio_1_	12 ton ha^−1^ compost + 2.4 ton ha^−1^ rock phosphate add with soil preparation for cultivation and Bio-N application was added 50 g/plot after 20 days of planting
3	MixBio_2_	12 ton ha^−1^ compost + 2.4 ton ha^−1^ rock phosphate add with soil preparation for cultivation and Bio-P application at the same time of application of Bio-N
4	MixBio_3_	12 ton ha^−1^ compost + 2.4 ton ha^−1^ rock phosphate add with soil preparation for cultivation and Bio-K application at the same time of application of Bio-N
5	Bio_1_	Bio-N application was added 50 g/plot after 20 days of planting
6	Bio_2_	Bio-P application at the same time of application of Bio-N
7	Bio_3_	Bio-K application at the same time of application of Bio-N

**Table 3:** Characterization of the different fertilizers and different irrigation scheduling used in the experiment

Treatments			Code		Characterzation											
Fertilization treatments	1	Chemical fertilizer	CF		Ammonium nitrate, Superphosphate and Potassium sulphate											
		Bio-Fertilizers			Content of bacteria											
	2	Bio-N (Microbine)	Bio_1_		*Azotobacter chroococcum, Asospirillum lipoferem, Asospirillum braselence and Azospirillum brasilense*											
		Bio-P (Phosphorine)	Bio_2_		*Bacillus sp., Bacillus megaterium and inegotherium phosphaticum*											
		Bio-K (Potassiumag)	Bio_3_		*Bacillus circulans*											
	3	Rock phosphate & Compost	Mix		Total macro- elements (mg kg^1^)				Total micro-elements (mg kg^1^)					O.M (%)	E.C (dSm^−1^) (1:5)	pH Susp. (1:2.5)
					N	P		K	Fe	Cu		Zn	Mn			
		Rock phosphate				248,000		100	2144	342		152	387		2.92	6.36
		compost			18,900	8300		10,600	1300	165		58	128	3.81	3.47	7.7
Irrigation scheduling	W1	No. of Irrigation (m^3^ha^−1^)	1	2	3	4	5	6	7	8	9	10	11	12	13	14
		Cut 1	320	323	324	326	327	330	330	331	335	338	342	344	345	345
		Cut 2	315	315	318	321	321	325	325	328	329	332	332	334	334	335
	W2	No. of Irrigation (m^3^ha^−1^)	1				2				3			4		
		Cut 1	1150				1166				1175			1188		
		Cut 2	1140				1155				1164			1173		

Each value in this table is the mean of 3 replicates

### Experimental design and irrigation treatments

The experiment was arranged in a randomized complete block design (RCBD) with a factorial arrangement consisting of two factors:

- Factor 1: Fertilization treatments (7 levels).
- Factor 2: Soil moisture regimes (2 levels):
  - W1: 100% of soil water content at field capacity FC (well-watered).
  - W2: 60% of soil water content at field capacity FC (water-stressed).

Each treatment was replicated three times. Each experimental plot measured 4 × 2.5 m (10 m^2^) and consisted of four rows spaced 50 cm apart, with plants spaced 35 cm within rows. Chemical fertilizer at 100% of soil water content at field capacity FC is the control treatment.

### Determination of soil moisture and irrigation scheduling

Soil field capacity and permanent wilting point were determined according to Walter [52] using the pressure membrane apparatus following Klute [53]. Undisturbed and disturbed soil samples were collected from depths of 0–90 cm prior to planting.

Soil moisture content was determined gravimetrically before and after irrigation. Irrigation scheduling was adjusted to maintain either 100% or 60% of field capacity by modifying irrigation frequency and water volume per irrigation event. The total irrigation water applied under each treatment is presented in (Table 3). The selection of 60% field capacity (FC) as a drought stress threshold was strategically chosen to represent moderate-to-severe water deficit common in arid and semi-arid regions. This level of stress is sufficient to trigger the plant's adaptive responses without reaching the permanent wilting point, allowing for a precise evaluation of how bio-organic amendments enhance physiological resilience and metabolic recovery in *Moringa oleifera.*

### Plant sampling and growth measurements

Plants were harvested twice during the growing season. The first cut was conducted 95 days after sowing, and the second cut was performed three months later. At each harvest, four plants were randomly selected from each plot and cut 10 cm above the soil surface.

The following parameters were recorded at each harvest: plant height, number of branches per plant, total plant dry weight, leaf dry weight per plant, and dry leaf yield (kg ha⁻^1^). Leaf samples were washed with distilled water, air-dried to constant weight, finely ground, and wet-digested using a sulfuric–perchloric acid mixture (3:1) for mineral analysis.

### Chemical analyses

Leaf nitrogen, phosphorus, potassium, and calcium contents were determined according to AOAC methods [54]. Soil pH, cation exchange capacity, organic matter, available nitrogen, phosphorus, and potassium were determined following standard protocols [55–58].

### Physiological and biochemical analyses

Physiological and biochemical analyses were conducted to assess the integrated physiological status of plants under different fertilization and irrigation treatments. Fresh leaf samples were collected from representative plants at the end of the growing season and were used for biochemical determinations. These analyses were not intended for harvest-based comparisons, and samples from different harvests were not analyzed separately.

#### Preparation of enzyme extracts

Fresh leaf tissue (0.5 g) was homogenized in chilled Tris–HCl buffer (0.05 M, pH 7.0) containing 3 mM MgCl₂ and 1 mM EDTA. The homogenate was centrifuged at 4 °C for 10 min at 5000 rpm, and the supernatant was used for enzyme activity assays.

Ethanolic extracts were prepared separately for non-enzymatic antioxidant determination by homogenizing fresh leaf tissue in 80% ethanol followed by centrifugation at 10,000 g for 5 min.

#### Biochemical determinations

Photosynthetic pigments, soluble proteins, proline, and ascorbic acid contents were determined according to Lichtenthaler [59], Lowry et al. [60], Bates et al. [61], and Jagota and Dani [62], respectively.

Catalase (CAT) activity was determined by following the consumption of H_2_O_2_ for 1 min according to Aebi [63], The reaction medium contained 50 mM potassium phosphate buffer (pH 7), 10 mM H_2_O_2_ and 100 μl of protein extract in a 4 ml volume for plant tissue. The decrease in absorbance at 240 nm was used to calculate the activity.

peroxidase (POD) activity was measured spectrophotometrically following the method of Tatiana et al. [64], with some modifications. The reaction mixture (4 ml) consisted of 30 mM potassium phosphate buffer (pH 7), 6.5 mM H_2_O_2_ and 100 μl enzyme extract for shoot and root. The increase in absorbance due to the formation of tetraguaiacol was measured at 470 nm.

Ascorbate peroxidase (APX) was assayed as described by Jiang and Zhang [65], but with some modifications. The rate of hydrogen peroxide-dependent oxidation of ascorbic acid was determined in reaction mixture contained in 4 ml of reaction medium containing 5 mM potassium phosphate buffer (pH 7), 1 mM H_2_O_2_ and 40 μl protein extract for shoot and root. The activity was determined by recording the decrease in A290 following the oxidation rate of ascorbic acid for 3 min.

The activity of phenylalanine ammonia lyase (PAL) was assayed according to the method described by Havir and Henson [66]. The reaction mixture containing 1 ml 0.04 M borate buffer (pH 8.7), 1 mg L-phenylalanine and 0.2 ml enzyme extract was incubated for 1 h at 37ºC and the reaction was terminated with 1 ml 0.5 N HCl. The mixture was centrifuged at 2000 rpm for 5 min before reading at 290 nm. The specific activity was expressed as unit mg^−1^ protein.

Polyphenol oxidase (PPO) activity was assayed by the method of Kumar and Khan [67]. Assay mixture for PPO contained 2 ml of 0.1 M phosphate buffer (pH 6), 1 ml of 0.1 M catechol and 0.2 ml of enzyme extract. This was incubated for 5 min at 25ºC, after which the reaction was stopped by adding 1 ml of 2.5 N H2SO4. The absorbance of the purpurogallin formed was read at 495 nm. To the blank 2.5 N H_2_SO_4_ was added of the zero time of the same assay mixture. PPO activity was expressed in UE mg^−1^ protein (UE = change in 0.1 absorbance min^−1^ mg ^−1^ protein).

Phenolics were determined according to Singleton and Rossi [68] with a slight modification using the Folin-Ciocalteu's phenol reagent. One hundred microliters of the extracts were mixed with 0.5 ml 2 N Folin-Ciocalteu's reagent and 2.5 ml of 20% Na_2_CO_3_. After 20 min. at room temperature, absorbance of samples was measured at 725 nm with a (EMC-11-UVspectrophotometer). The results were expressed as gallic acid equivalents as μg g^−1^ fresh matter using Molar Coefficient of 120 µg^−1^ cm^−1^ ml^−1^.

Total flavonoid content measurement was based on the method described by Moreno et al. [69] with a slight modification. An aliquot of 100 µL of Tris- solution containing 1 mg of extract was added to test tubes containing 0.1 ml of 10% aluminum nitrate, 0.1 ml of a 1 M potassium acetate solution and 3.8 ml of methanol. After 40 min at room temperature, the absorbance is measured at 415 nm. Qurecetine was used as standard. The result was represented as µg g^−1^ FW. Total antioxidant capacity and reducing power were determined using the methods of Prieto et al. [70] and Oyaizu [71].

### Statistical analysis

Data were subjected to two-way analysis of variance (ANOVA) to evaluate the effects of fertilization, irrigation, and their interaction using SPSS software (version 16). Mean comparisons were performed using Duncan’s multiple range test at p ≤ 0.05.

Multivariate analyses, including principal component analysis (PCA), heatmap clustering, and correlation analysis, were conducted using R software (RStudio). Data were standardized prior to analysis.

## Results

### Effect of bio-organic amendments on soil physicochemical characteristics

The synergistic interaction of bio-organic fertilizers and irrigation systems had major effects on soil chemical properties such as organic matter content (OM), cationic exchange capacity (CEC), soil pH, and available macronutrients (N, P, and K) (Table 4). Overall, the fertilization system had greater effects on soil OM% than irrigation rates. All bio-organic fertilizers significantly enriched the soil OM pool. Clearly, MixBio_2_ recorded the highest soil OM% under both optimal (2.61%) and drought-stressed (2.12%) conditions, meanwhile (CF) control exhibited had the lowest values of soil OM%.

**Table 4:** Soil properties (O.M., C.E.C., pH Susp., Ava-N, Ava-P and Ava-K) as affected by two irrigation scheduling and different fertilizers treatments

Treatments		O.M (%)	C.E.C (cmolc kg^−1^)	pH Susp. (1:2.5)	Available-N (mg/kg)	Available -P (mg/kg)	Available -K (mg/kg)
**Irrigationscheduling**	**fertilizers**						
W_1_	CF	2.19±0.035^f^	14.21±0.346^i^	8.12±0.006^ab^	22.73±0.404^j^	23.16±0.173^d^	167.51±0.115^l^
	MixBio_1_	2.56±0.052^b^	15.95±0.462^a^	7.36±0.012^e^	26.28±0.462^a^	21.42±0.012^h^	174.66±0.173^i^
	MixBio_2_	2.61±0.035^a^	15.73±0.412^b^	7.86±0.012^d^	24.16±0.462^e^	25.88±0.231^a^	216.45±0.173^e^
	MixBio_3_	2.49±0.035^c^	15.50±0.412^de^	8.07±0.006^b^	23.17±0.404^i^	23.33±0.173^d^	281.57±0.115^a^
	Bio_1_	2.25±0.040^e^	15.24±0.346^c^	7.84±0.012^d^	24.91±0.462^c^	20.25±0.115^i^	173.18±0.115^j^
	Bio_2_	2.50±0.035^bc^	15.02±0.412^hg^	7.93±0.006^cd^	23.20±0.412^g^	24.87±0.115^b^	203.15±0.173^g^
	Bio_3_	2.42±0.029^d^	14.82±0.288^h^	8.10±0.012^b^	22.83±0.462^i^	22.50±0.173^f^	263.93±0.173^c^
W_2_	CF	1.36±0.035^l^	14.15±0.462^j^	8.08±0.006^b^	22.14±0.462^l^	22.56±0.115^ef^	166.38±0.231^l^
	MixBio_1_	2.05±0.046^g^	15.53±0.404^d^	7.43±0.012^e^	25.87±0.404^b^	21.16±0.115^h^	173.35±0.115^ij^
	MixBio_2_	2.12±0.040^f^	15.44±0.346^ef^	7.90±0.006^cd^	23.86±0.346^f^	25.56±0.173^b^	214.32±0.173^f^
	MixBio_3_	1.98±0.035^h^	15.08±0.346^g^	8.14±0.006^ab^	22.87±0.404^h^	22.83±0.173^e^	280.04±0.115^b^
	Bio_1_	1.65±0.040^k^	15.03±0.404^hg^	7.87±0.006^d^	24.39±0.462^d^	19.88±0.115^j^	170.82±0.115^k^
	Bio_2_	1.90±0.035^i^	15.00±0.346^h^	7.98±0.006^c^	22.62±0.346^j^	24.46±0.173^c^	201.58±0.173^h^
	Bio_3_	1.72±0.029^j^	14.52±0.288^i^	8.19±0.006^a^	22.33±0.412^k^	22.14±0.173^g^	262.22±0.173^d^
F value		1.728^NS^	2.229^NS^	1.00^NS^	0.131^NS^	1.002^NS^	0.611^NS^
*P* value		0.151	0.000	0.000	0.991	0.443	0.72

Different superscript litters are significantly different at *p* < 0.05, (mean ± SE; *n* = 3). Two-way ANOVA, Duncan's test at *P* < 0.05 level for interaction

*Abbreviations*; *W*_1_ & *W*_2_ Two irrigation scheduling, *CF* Chemical fertilizers, *Bio*_1_ Biofertilizer Microbine, *Bio*_2_ Biofertilizer Phosphorine, *Bio*_3_ Biofertilizer Potassiumag, *MixBio*_1_ 12 ton ha^−1^ compost + 2.4 ton ha^−1^ rock phosphate with Bio_1,_ *MixBio*_2_ 12 ton ha^−1^ compost + 2.4 ton ha^−1^ rock phosphate with Bio_2,_ *MixBio*_3_ 12 ton ha^−1^ compost + 2.4 ton ha^−1^ rock phosphate with Bio_3_

**high significant, *significant, *NS* Non-significant

CEC values of soil were also highly influenced by treatment, showing significant interaction between irrigation levels and fertilizer combinations. MixBio treatment groups 1, 2, and 3 had higher CEC values when irrigated optimally (W_1_) than when subjected to drought conditions (W_2_).

In both irrigation systems (W1 and W2), the MixBio treatments showed a superior effect to improve nutrients availability as compared with either individual bio-treatments or chemical fertilization (CF) control. Available N was largely affected by MixBio1 treatment, which recorded the highest value (26.28 mg/kg). For available P, MixBio_2_ performed better than other treatments and sustained the highest concentration (about 25.7 mg/kg) under both water stress (W2) and optimal irrigation (W1). Available K also recorded a significant enhancement, particularly with MixBio_3_, which increased K concentration from 167.51 mg/kg to 281.57 mg/kg.

Soil pH values were significantly lowered by the addition of fertilizer combinations under treatment W_1_ than under treatment W_2_. In particular, a significant reduction in the soil pH was observed in MixBio1&2-treated plots, dropped from initial soil baseline 8.05 to about 7.86–7.36 under well water condition (W1), and 7.43–7.9 under drought stress (W2), which was a noticeable change. Soil acidification, probably driven by microbial organic acid secretion, could be closely linked to a highly significant rise in available phosphorus (Ava-P) in the MixBio2 treatment (Phosphorine + Rock Phosphate + Compost). additionally, the levels of organic matter (OM) and available nitrogen (Ava-N) both exhibited a synergistic enhancement, which implying to the positive effect of incorporating compost as stable source of carbon that protected plants from the water-deficit stress (W2) negative impact.

The overall efficiency of different treatments in improving soil dynamics followed a decreasing order: MixBio_3_ > Bio_3_ > MixBio_2_ > Bio_2_ > MixBio_1_ > Bio_1_ > CF.

### Effect of treatments on growth parameters and yield of plant

The growth attributes of *Moringa oleifera* exhibited a significant decline under drought stress (60% FC); however, the integrated application of bio-organic amendments effectively buffered this reduction, sustaining higher biomass such as fresh weight, dry weight, plant height (Table 5), dry leaves weight, and dry leaves yield (Table 6), compared to the untreated water-stressed plants.

**Table 5:** Fresh weight, Dry weight, No of branches and plant height of *Moringa oleifera* as affected by two irrigation scheduling and different fertilizers treatment

Treatments		Fresh weight (g\plant)		Dry weight (g\plant)		No of branches		Plant height (cm)	
Irrigation scheduling	Fertilizers	cut 1	cut 2	cut 1	cut 2	cut 1	cut 2	cut 1	cut 2
W_1_	CF	182.5±1.391^b^	451.9±1.795^b^	38.8±0.462^c^	138.4±0.724^b^	16.7±0.173^c^	16.0±0.115^d^	120.0±0.866^d^	130.0±0.745^b^
	MixBio_1_	211.0±0.704^a^	816.9±1.062^a^	44.2±0.173^a^	203.0±0.294^a^	18.3±0.173^a^	19.0±0.115^a^	150.0±0.692^a^	130.0±0.522^a^
	MixBio_2_	107.5±1.168^h^	250.6±1.856^g^	21.3±0.254^e^	55.5±0.758^j^	12.7±0.231^g^	12.0±0.173^h^	145.3±0.641^b^	81.7±0.942^f^
	MixBio_3_	110.1±0.826^g^	300.3±1.341^f^	22.7±0.185^d^	82.9±0.536^h^	16.0±0.115^d^	15.3±0.115^e^	107.7±0.491^f^	116.7±0.667^c^
	Bio_1_	100.0±0.462^j^	203.6±1.258^i^	20.2±0.173^f^	96.9±0.522^f^	8.3±0.115^i^	9.0±0.173^i^	63.3±0.462^l^	75.0±0.577^g^
	Bio_2_	113.0±0.724^f^	239.4±1.391^h^	18.8±0.115^g^	67.9±0.603^i^	13.3±0.173^f^	13.0±0.115^g^	124.0±0.693^c^	105.7±0.511^d^
	Bio_3_	90.1±0.522^k^	200.8±0.462^j^	15.8±0.173^h^	41.7±0.231^l^	14.3±0.115^e^	14.0±0.173^f^	94.0±0.577^h^	85.0±0.462^e^
W_2_	CF	81.5±0.294^l^	201.7±0.811^i^	14.5±0.115^i^	51.9±0.322^k^	10.0±0.173^h^	9.0±0.115^i^	100.0±0.811^g^	49.0±0.577^h^
	MixBio_1_	58.8±0.522^m^	162.9±0.354^k^	11.3±0.115^j^	26.1±0.231^n^	13.3±0.115^f^	13.0±0.173^g^	90.0±0.577^i^	74.0±0.522^g^
	MixBio_2_	105.6±0.866^i^	382.0±0.942^d^	16.6±0.173^h^	106.1±0.811^e^	17.3±0.231^b^	17.7±0.231^b^	88.3±0.866^j^	134.3±0.536^a^
	MixBio_3_	42.5±0.354^n^	145.2±0.522^l^	9.7±0.115^k^	33.9±0.294^m^	13.0±0.173^f^	13.3±0.115^g^	100.3±0.693^g^	75.0±0.511^g^
	Bio_1_	140.0±0.693^d^	240.3±1.258^h^	40.00.173^b^	120.0±0.491^d^	13.3±0.115^f^	14.0±0.173^f^	80.0±0.577^k^	85.0±0.462^e^
	Bio_2_	158.1±0.536^c^	308.4±0.745^e^	40.2±0.115^b^	88.4±0.462^g^	16.7±0.173^c^	17.0±0.115^c^	111.7±0.811^e^	130.0±0.432^b^
	Bio_3_	130.0±0.462^e^	446.9±0.866^c^	22.4±0.115^d^	136.7±0.522^c^	17.3±0.173^b^	17.0±0.231^c^	120.0±0.577^d^	130.0±0.641^b^
F value		19,390.3^**^	202,429^**^	935.4^**^	19,150.9^**^	59.9^**^	0.747^NS^	2628.8^**^	5705.2^**^
*P* value		0.000	0.000	0.000	0.000	0.000	0.617	0.000	0.000

Different superscript litters are significantly different at *p* < 0.05, (mean ± SE; *n* = 3). Two-way ANOVA, Duncan's test at *P* < 0.05 level for interaction

*Abbreviations*; *W*_1_ & *W*_2_ Two irrigation scheduling, *CF* Chemical fertilizers, *Bio*_1_ Biofertilizer Microbine, *Bio*_2_ Biofertilizer Phosphorine, *Bio*_3_ Biofertilizer Potassiumag, *MixBio*_1_ 12 ton ha^−1^ compost + 2.4 ton ha^−1^ rock phosphate with Bio_1,_ *MixBio*_2_ 12 ton ha^−1^ compost + 2.4 ton ha^−1^ rock phosphate with Bio_2,_ *MixBio*_3_ 12 ton ha^−1^ compost + 2.4 ton ha^−1^ rock phosphate with Bio_3_

**high significant, *significant, *NS* Non-significant

**Table 6:** Dry leaves weight and yield dry leaves of *Moringa oleifera* as affected by two irrigation scheduling and different combinations fertilizers treatment

Treatments		Dry leaves weight (g\plant)		Mean	Yield dry leaves (Kg/ha)		Mean
**Irrigation scheduling**	**Fertilizers**	**cut 1**	**cut 2**		**cut 1**	**cut 2**	
W_1_	CF	14.6±0.201^b^	38.8±0.231^a^	26.70	582.0±0.115^b^	1547.2±0.231^a^	1064.60
	MixBio_1_	15.9±0.173^a^	26.3±0.115^c^	21.10	628.7±0.081^a^	1061.1±0.462^c^	844.90
	MixBio_2_	6.2±0.075^i^	10.3±0.173^gh^	8.25	248.9±0.046^i^	410.5±0.231^g^	329.70
	MixBio_3_	8.6±0.006^e^	17.1±0.231^e^	12.85	342.3±0.404^e^	688.4±0.173^e^	515.35
	Bio_1_	2.2±0.012^k^	6.0±0.173^j^	4.10	86.3±0.04^l^	245.3±0.115^h^	165.80
	Bio_2_	8.2±0.012^f^	10.4±0.115^gh^	9.30	326.4±0.346^f^	411.9±0.692^g^	369.15
	Bio_3_	9.0±0.006^d^	11.0±0.231^g^	10.00	359.9±0.385^d^	438.3±0.522^f^	399.10
W_2_	CF	13.0±0.075^c^	30.0±0.173^b^	21.50	525.3±0.404^c^	1204.0±0.231^b^	864.65
	MixBio_1_	5.4±0.081^j^	7.3±0.115^i^	6.35	216.0±0.231^j^	290.3±0.173^g^	253.15
	MixBio_2_	7.7±0.001^g^	26.2±0.017^c^	16.95	311.5±0.231^g^	1053.1±0.013^c^	682.30
	MixBio_3_	4.4±0.001^j^	10.6±0.231^gh^	7.50	175.1±0.173^k^	424.8±0.173^ef^	299.95
	Bio_1_	7.3±0.001^h^	11.0±0.288^g^	9.15	290.3±0.115^h^	434.0±0.231^ef^	362.15
	Bio_2_	8.9±0.006^d^	15.4±0.02^f^	12.15	350.9±0.017^e^	613.7±0.017^f^	482.30
	Bio_3_	9.0±0.012^d^	20.6±0.462^d^	14.80	366.7±0.412^d^	808.8±0.412^d^	587.75
F value		77.7^**^	434.6^**^		2433.6^**^	1879.2^**^	
*P* value		0.000	0.000		0.000	0.000	

Different superscript litters are significantly different at *p* < 0.05, (mean ± SE; *n* = 3). Two-way ANOVA, Duncan's test at *P* < 0.05 level for interaction

*Abbreviations*; *W*_1_ & *W*_2_ Two irrigation scheduling, *CF* Chemical fertilizers, *Bio*_1_ Biofertilizer Microbine, *Bio*_2_ Biofertilizer Phosphorine, *Bio*_3_ Biofertilizer Potassiumag, *MixBio*_1_ 12 ton ha^−1^ compost + 2.4 ton ha^−1^ rock phosphate with Bio_1,_*MixBio*_2_ 12 ton ha^−1^ compost + 2.4 ton ha^−1^ rock phosphate with Bio_2,_ *MixBio*_3_ 12 ton ha^−1^ compost + 2.4 ton ha^−1^ rock phosphate with Bio_3_

**high significant, *significant, *NS* Non-significant

According to the data presented in (Table 5), biofertilizers Bio1, Bio2, and Bio3 significantly increased the fresh and dry weights of *Moringa oleifera* plants in both the first cut (Cut 1) and the second cut (Cut 2) under drought stress (W2), compared with chemical fertilizer (CF) under drought stress (W2). While MixBio_2_ showed similar response in the second cut only. On the other hand, chemical fertilizer, MixBio_1_ and MixBio_3_ could not improve the fresh and dry biomass under drought stress as compared to its corresponding treatments under (W1).

Chemical fertilizer, MixBio_1_and MixBio_3_ under drought stress (W_2_) reduced number of branches as compared to corresponding treatments under optimal irrigation (W_1_) (Table 5). Biofertilizers MixBio_1_ and MixBio_3_ treatments exhibited higher number of branches when compared to chemical fertilizer. Bio-fertilizing Moringa plants with MixBio_2_, Bio_1_, Bio_2_ and Bio_3_ increased the number of branches despite of the drought stress as compared to CF control and corresponding treatments under W1. These responses were observed in the first and the second cut.

According to plant height data (Table 5) at the first cut, it was clear that Bio_2_ and Bio_3_ under water level (W_1_) induced the plant height as compared to CF control. While in the second cut, all the fertilizers induced plant height at W_1_ as compared to corresponding CF control.

In terms of dry leaf yield (Table 6), it’s interesting to note that while the chemical fertilizer (CF) sustained a marginal numerical lead in both irrigation regimes (W1&2), the MixBio2 treatment didn’t show a statistically significant difference in many cases, particularly when the plants subjected to drought stress (W2). This shows that the integrated bio-organic approach (PGPR + compost + rock phosphate) can be as productive as conventional chemical systems. This performance, along with the improvements in soil health that were mentioned earlier, makes the MixBio strategy a strong and long-lasting option for growing *M. oleifera* in dry regions.

### Effect of treatments on physiological and biochemical parameters of plant

To reflect a complete understanding of the plant’s ability to adapt, its leaf photosynthetic pigments, mineral nutrient status (Table 7), and antioxidant defense mechanisms (Table 8) during the second harvest were assessed. This sampling point was strategically chosen to exhibit the cumulative physiological response and the highest level of adaptive metabolic changes after prolonged exposure to drought stress (W2). in order to confirming a strong connection and correlation between the soil’s nutritional status and the plant’s internal physiological and biochemical defense systems. A synergistic effect was observed between bio-fertilizers and organic amendments in protecting the photosynthetic apparatus, as evidenced by the significantly higher chlorophyll and carotenoid contents in treated plants under both optimal and stressed conditions. Treating moringa trees with two water levels resulted in highly significant change in chlorophyll a content (Table 7). Biofertlizer Bio_1_ showed the highest level of ch.a content under drought stress (W2) as compared to corresponding treatment under (W_1_), and even when compared to chemical fertilizer FC control. Other fertilizers treatment resulted in similar response. It can be arranged as Bio_1_ > MixBio_2_ > Bio_2_ > Bio_3_ > MixBio_1_ > MixBio_3_. The water levels (W_1_ & W_2_) affected chlorophyll b content highly significantly. Bio_1_ fertilizer treatment enhanced the content of ch.b under drought stress (W2) as compared to chemical fertilizer FC control and corresponding treatment under optimal irrigation (W_1_). Other fertilizer treatments such as Bio_3_, Bio_2_, MixBio_2_, MixBio_3_ and MixBio_1_ also enhanced ch.b content when compared to CF control (Table 7).

**Table 7:** Photosynthetic pigments (ch.a, Ch.b, Carot.), some elements contents (K, Ca, P, N) and soluble proteins content of *Moringa oleifera* leaves as affected by two irrigation scheduling and different combinations fertilizers treatment

Treatments		Chl.a. mg g^−1^FW	Chl.b. mg g^−1^ FW	Carot mg g^−1^ FW	K mg g^−1^ DW	Ca mg g^−1^ DW	P mg g^−1^ DW	N g/100 g DW	Protein mg g^−1^ DW
**Irrigation scheduling**	**Fertilizers**								
W_1_	CF	88.5±0.007^j^	8.2±0.003^l^	40.2±0.003^j^	29.8±0.007^e^	9.0±0.006^c^	4.3±0.003^i^	2.5±0.013^c^	118.3±0.081^i^
	MixBio_1_	134.8±0.007^e^	9.0±0.007^k^	50.2±0.007^g^	23.5j±0.01^k^	7.5±0.007^e^	5.6±0.007^g^	2.4±0.013^d^	134.1±0.081^f^
	MixBio_2_	107.7±0.001^h^	18.0±0.006^g^	44.4±0.006^h^	18.5±0.012^l^	5.8±0.006^g^	5.4±0.001^g^	1.9±0.012^e^	201.9±0.075^a^
	MixBio_3_	106.5±0.013^h^	17.4±0.012^h^	44.5±0.012^h^	26.0±0.01^h^	6.5±0.006^f^	6.2±0.013^e^	2.1±0.012^e^	139.3±0.081^e^
	Bio_1_	106.1±0.006^h^	9.3±0.006^k^	43.3±0.006^i^	36.3±0.012^b^	9.5±0.006^c^	5.9±0.006^f^	2.4±0.012^d^	62.9±0.075^m^
	Bio_2_	147.2±0.013^b^	25.1±0.001^c^	57.9±0.017^b^	33.5±0.017^d^	8.0±0.013^d^	8.3±0.013^a^	2.0±0.017^e^	179.0±0.081^b^
	Bio_3_	139.1±0.01^d^	24.6±0.007^d^	54.6±0.02^d^	47.0±0.007^a^	7.3±0.01^e^	7.0±0.01^c^	2.4±0.007^d^	174.0±0.462^c^
W_2_	CF	80.0±0.007^k^	6.0±0.001^m^	50.4±0.017^g^	27.3±0.013^g^	8.0±0.017^d^	8.3±0.007^a^	2.7±0.017^a^	80.2±0.081^k^
	MixBio_1_	120.5±0.001^g^	11.0±0.001^j^	51.9±0.001^f^	23.5±0.006^j^	9.3±0.012^c^	4.3±0.001^i^	1.9±0.006^e^	52.3±0.075^n^
	MixBio_2_	146.8±0.013^b^	20.8±0.007^f^	58.2±0.007^b^	33.5±0.01^d^	7.3±0.007^e^	7.4±0.013^b^	2.4±0.01^d^	131.1±0.081^g^
	MixBio_3_	96.3±0.006^i^	15.8±0.006^i^	50.4±0.006^g^	28.0±0.012^f^	11.5±0.006^a^	6.2±0.006^e^	2.6±0.012^b^	111.7±0.075^j^
	Bio_1_	152.1±0.013^a^	32.1±0.012^a^	59.3±0.012^a^	28.5±0.01^f^	6.8±0.006^f^	6.6±0.013^d^	2.7±0.012^a^	70.7±0.081^l^
	Bio_2_	145.6±0.006^c^	23.0±0.006^e^	55.7±0.006^c^	34.5±0.012^c^	10.5±0.006^b^	6.8±0.006^d^	2.7±0.012^a^	144.4±0.075^d^
	Bio_3_	134.2±0.013^f^	26.7±0.001^b^	53.7±0.017^e^	24.8±0.017^i^	11.5±0.013^a^	4.9±0.013^h^	2.4±0.017^d^	128.5±0.081^h^
F value		818.6^**^	1.465^NS^	154.6^**^	0.944^NS^	0.642^NS^	100.13^**^	4.03^**^	1509.3^**^
*P* value		0.000	0.226	0.000	0.48	0.696	0.000	0.005	0.000

Different superscript litters are significantly different at *p* < 0.05, (mean ± SE; *n* = 3). Two-way ANOVA, Duncan's test at *P* < 0.05 level for interaction

*Abbreviations*; *W*_1_ & *W*_2_ Two irrigation scheduling, *CF* Chemical fertilizers, *Bio*_1_ Biofertilizer Microbine, *Bio*_2_ Biofertilizer Phosphorine, *Bio*_3_ Biofertilizer Potassiumag, *MixBio*_1_ 12 ton ha^−1^ compost + 2.4 ton ha^−1^ rock phosphate with Bio_1,_ *MixBio*_2_ 12 ton ha^−1^ compost + 2.4 ton ha^−1^ rock phosphate with Bio_2,_ *MixBio*_3_ 12 ton ha^−1^ compost + 2.4 ton ha^−1^ rock phosphate with Bio_3_

**high significant, *significant, *NS* Non-significant

**Table 8:** Total antioxidants, Reducing power and non-enzymatic antioxidants (Flavonoids, Phenolics, proline, and Vit. C) of *Moringa oleifera* leaves as affected by two irrigation scheduling and different combinations fertilizers treatment

Treatments		Total antioxidants Abs. (at 695 nm ml^−1)^	Reducing power µg g^−1^ FW	Flavonoids mg g^−1^ FW	Phenolics µg g^−1^ FW	Proline mg g^−1^ DW	Vitamin C mg g^−1^ FW
**Irrigation scheduling**	**Fertilizers**						
W_1_	CF	33.098±0.404^f^	0.167±0.007^j^	1.071±0.064^j^	1.922±0.115^j^	1.128±0.003^f^	1.849±0.294^h^
	MixBio_1_	30.247±0.231^g^	0.15±0.01^k^	1.008±0.115^k^	1.340±0.003^m^	1.981±0.017^a^	1.821±0.462^i^
	MixBio_2_	40.059±0.231^e^	0.194±0.012^h^	1.189±0.173^i^	1.911±0.006^j^	1.550±0.017^b^	2.431±0.412^c^
	MixBio_3_	46.926±0.173^c^	0.227±0.012^g^	1.286±0.231^h^	1.748±0.012^l^	1.148±0.02^e^	1.896±0.462^g^
	Bio_1_	46.848±0.115^c^	0.278±0.006^c^	1.822±0.231^c^	2.847±0.012^d^	0.502±0.173^l^	1.315±0.412^l^
	Bio_2_	63.836±0.231^a^	0.354±0.081^a^	2.267±0.173^a^	6.371±0.075^a^	1.354±0.001^d^	2.828±0.288^a^
	Bio_3_	45.145±0.173^d^	0.24±0.046^f^	1.580±0.404^f^	2.299±0.04^h^	0.782±0.346^j^	2.478±0.385^b^
W_2_	CF	41.157±0.231^e^	0.150±0.404^l^	0.996±0.231^k^	2.031±0.231^i^	0.781±0.173^j^	1.974±0.115^f^
	MixBio_1_	45.000±0.115^d^	0.224±0.007^g^	1.781±0.173^d^	2.953±0.173^c^	0.492±0.115^m^	1.303±0.231^m^
	MixBio_2_	41.356±0.173^e^	0.267±0.006^d^	1.668±0.231^e^	2.586±0.012^f^	1.085±0.173^g^	1.601±0.288^j^
	MixBio_3_	56.654±0.115^b^	0.345±0.006^b^	2.039±0.173^b^	2.736±0.012^e^	0.964±0.115^i^	2.011±0.231^e^
	Bio_1_	33.973±0.173^f^	0.189±0.001^i^	1.355±0.017^g^	1.820±0.017^k^	0.600±0.173^k^	1.234±0.231^n^
	Bio_2_	39.991±0.115^e^	0.247±0.001^e^	1.583±0.173^f^	2.328±0.231^g^	1.414±0.173^c^	1.375±0.115^k^
	Bio_3_	28.161±0.173^g^	0.119±0.006^m^	0.780±0.231^l^	4.355±0.231^b^	1.045±0.231^h^	2.327±0.115^d^
F value		410.5^**^	8.6^**^	39.12^**^	61.24^**^	3.912^**^	9.3^**^
*P* value		0.000	0.000	0.000	0.000	0.006	0.000

Different Superscript litters are significantly different at *p* < 0.05, (mean ± SE; *n* = 3). Two-way ANOVA, Duncan's test at *P* < 0.05 level for interaction

*Abbreviations*; *W*_1_ & *W*_2_ Two irrigation scheduling, *CF* Chemical fertilizers, *Bio*_1_ Biofertilizer Microbine, *Bio*_2_ Biofertilizer Phosphorine, Bio_3_ Biofertilizer Potassiumag, *MixBio*_1_ 12 ton ha^−1^ compost + 2.4 ton ha^−1^ rock phosphate with Bio_1,_ *MixBio*_2_ 12 ton ha^−1^ compost + 2.4 ton ha^−1^ rock phosphate with Bio_2,_ *MixBio*_3_ 12 ton ha^−1^ compost + 2.4 ton ha^−1^ rock phosphate with Bio_3_

**high significant, *significant, *NS* Non-significant

The Carotenoid content of *Moringa oleifera* is presented in (Table 7). The data exhibited highly significant change as affected by the two water levels. Fertilizer treatments Bio_1_ and MixBio_2_ enhanced carotenoids content under drought condition (W_2_) as compared to chemical fertilizer control and corresponding treatments under optimal irrigation water (W_1_).

Data in (Table 7) revealed that bio-organic treatments induced differential responses in macronutrient accumulation two water levels (W1&2). While drought stress (W_2_) generally reduced nutrient concentrations, certain treatments like Bio_1_ and Bio_2_ demonstrated a capacity to maintain N and K^+^ levels comparable to CF control. Specifically, there was a highly significant difference in N content between the two water levels. While MixBio_1_ fertilizer under drought stress (W2) resulted in the lowest N content, Bio_1_ and Bio_2_ induced N content to levels similar to those observed in chemical fertilizer under water stress (W_2_). Chemical fertilizer control showed the highest nitrogen content value under W_1_ (Table 7), followed by MixBio_1_, Bio_3_ and Bio_1_. While MixBio_2_, Bio_2_ and MixBio_3_ showed the lowest values. From the data represented in (Table 7) there was a highly significant difference in K^+^ content as affected by the two water levels. Obviously Bio_3_ increased the K^+^ content to the highest level under optimal irrigation (W_1_). While Bio_2_, MixBio_2_, Bio_1_ and MixBio_3_ induced K^+^ content as compared to the chemical fertilizer treatment under water stress treatment. There was a highly significant difference in calcium content between the two water levels as shown in (Table 7). It was observed that MixBio_3_, Bio_3_, Bio_2_ and MixBio_1_ raised the calcium content under drought stress as compared to the corresponding chemical fertilizer. On the other hand, reduction in Ca^+2^ content was observed under MixBio_2_ and Bio_1_ when compared to chemical fertilizer at W_2_. There is a highly significant difference observed in phosphorous content between the two water levels (W_1_ & W_2_). The highest phosphorus content was observed at chemical fertilizer under water stress level, followed by MixBio_2_, Bio_2_ and Bio_1_. While MixBio_1_ and Bio_3_ exhibited the lowest phosphorus, contents under water stress treatment (Table 7).

It was obvious from the data obtained herein (Table 7) that water stress level (W_2_) reduced the content of soluble proteins under almost all fertilizers as compared to the corresponding treatments under optimal irrigation (W_1_). The biofertilizer Bio_1_ is the only exception that showed slight induction in soluble proteins under water stress level as compared to corresponding treatment under optimal irrigation W_1_.

From the data presented in (Table 8) there was a significant interaction between water levels and different fertilizers. Application of different fertilizers, particularly Bio_2_ and MixBio_3_ induced significantly the total antioxidants to the highest levels under W_1_ and W_2_, respectively. The lowest value of total antioxidants was recorded at Bio_3_ under W_2_. Generally, MixBio_1_ and MixBio_3_ induced the total antioxidants under drought stress (W_2_) compared to chemical fertilizer control and to its corresponding treatments under (W_1_).

It was obvious from the data represented in the (Table 8) that irrigating *Moringa oleifera* trees with two different water levels, control (W_1_) and drought treatment (W_2_) resulted in highly significant difference in the reducing power content and highly significant interaction was observed between water levels and fertilizers. Biofertilizers MixBio_3_, MixBio_2_ and MixBio_1_ enhanced the reducing power content to the highest level under drought condition (W_2_) as compared to chemical fertilizer control and corresponding treatments under (W_1_). While the two biofertilizers Bio_2_ and Bio_1_ showed a higher reducing power ability under W_2_ as compared to chemical fertilizer control, but lower content as compared corresponding treatments under W_1_.

Highly significant interaction was observed between water levels and fertilizers and significant difference in flavonoids content was observed between the two water levels (W_1_ and W_2_). Biofertilizers MixBio_3_, MixBio_1_ and MixBio_2_, induced the production of flavonoids under drought stress W_2_ as compared to the corresponding treatments under optimal irrigation (W_1_) as shown in (Table 8), while the rest of fertilizers did not exhibit the same response.

From the data obtained in (Table 8), there was high significance in phenolics content between the two water levels, different biofertilizers, and highly significant interaction between water levels and biofertilizers. Drought stress induced phenolics production under Bio_3_, MixBio_1_, MixBio_3_, and MixBio_2_, respectively, more than chemical fertilizer control and corresponding treatments under (W_1_). While biofertilizers Bio_2_ and Bio_1_ induced phenolics production under control water level (W_1_) more than corresponding treatments under drought stress (W_2_).

Proline content (Table 8) showed highly significant difference between the two water levels (W_1_ &W_2_). Highly significant difference was observed among different fertilizers and highly significant interaction between water levels and fertilizers treatments was also detected. The biofertilizers MixBio_1_, MixBio_2_ and MixBio_3_ in addition to the chemical fertilizer CF showed pronounced reduction in proline content under drought stress level (W_2_) as compared to corresponding treatments under optimal irrigation (W_1_). While other biofertilizers Bio_3_, Bio_1_, and Bio_2_, respectively, showed induction in proline content under drought stress (W_2_) as compared to corresponding treatments under (W_1_). The biofertilizers MixBio_3_ and CF treatment induced slightly the production of ascorbic acid (vitamin C) under drought stress level (W_2_) as compared to corresponding treatments under optimal water level (W_1_) (Table 8). While other biofertilizers showed reduction in ascorbic acid production under drought stress level. On the other hand, Bio_2_, MixBio,_2_ and MixBio_1_, respectively, induced ascorbic acid production under optimal water level (W_1_).

**Fig. 1:**
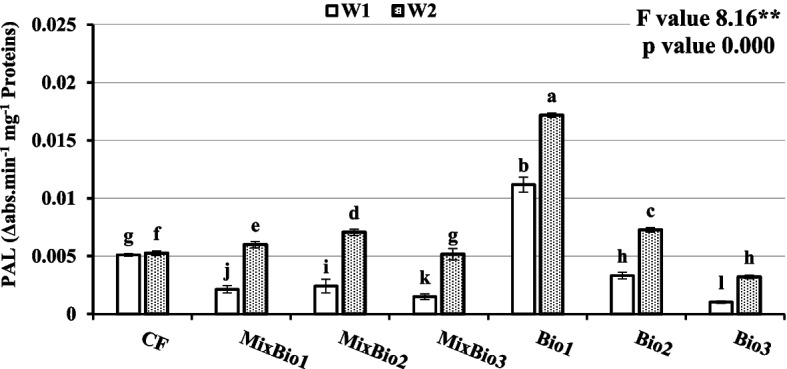
The activity of Phenylalanine ammonia lyase enzyme (PAL) of *Moringa oleifera* plants as affected by two water levels control (W_1_) and water stress (W_2_), and different fertilizers. Different litters are significantly different at *p* < 0.05, (mean ± SE; *n* = 3). Interaction F value and *p* value at *P* < 0.05 level. ** high significant, * significant, NS non-significant

**Fig. 2:**
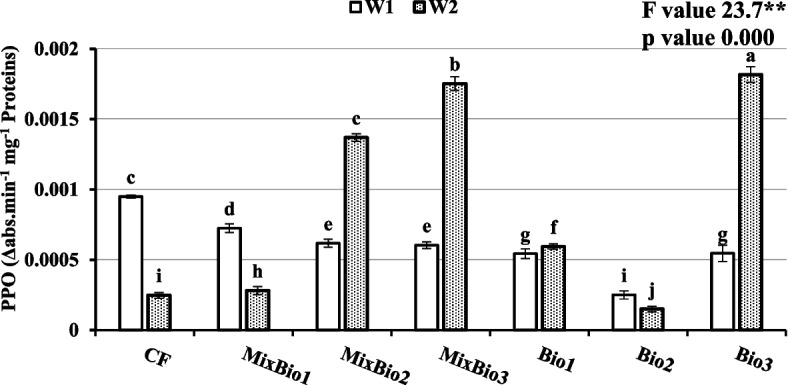
The activity of polyphenol oxidase enzyme (PPO) of *Moringa oleifera* plants as affected by two water levels control (W_1_) and water stress (W_2_), and different fertilizers. Different litters are significantly different at *p* < 0.05, (mean ± SE; *n* = 3). Interaction F value and *p* value at *P* < 0.05 level. ** high significant, * significant, NS non-significant

The activity of PAL enzyme is presented in Fig. 1. Bio_1_ treatment under W_2_ and W_1_ respectively, exhibited the highest value of PAL activity as compared to other fertilizers and chemical fertilizer. Water stress treatment (W_2_) induced PPO activity under Bio_3_, MixBio_3_, and MixBio_2_, respectively, as compared to chemical fertilizer control and corresponding treatments at W_1_ Fig. 2. While Bio_2_, CF, and MixBio_1_ under W_2_ showed the lowest activity of PPO as compared to other fertilizers under W_2_ treatments.

**Fig. 3:**
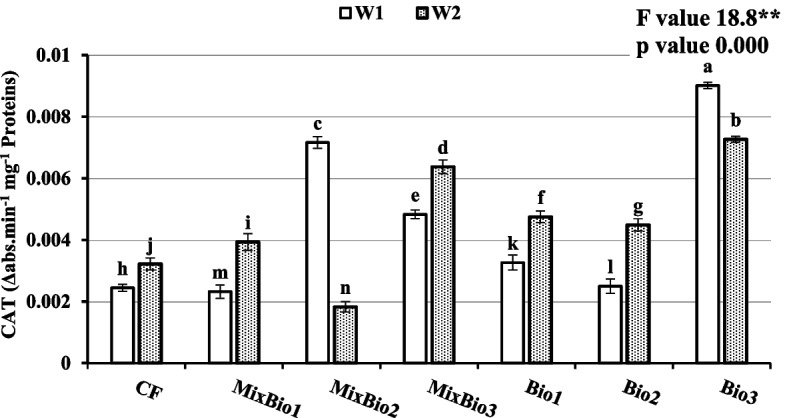
The activity of catalase enzyme (CAT) of *Moringa oleifera* plants as affected by two water levels control (W_1_) and water stress (W_2_), and different fertilizers. Different litters are significantly different at *p* < 0.05, (mean ± SE; *n* = 3). Interaction F value and *p* value at *P* < 0.05 level. ** high significant, * significant, NS non-significant

**Fig. 4:**
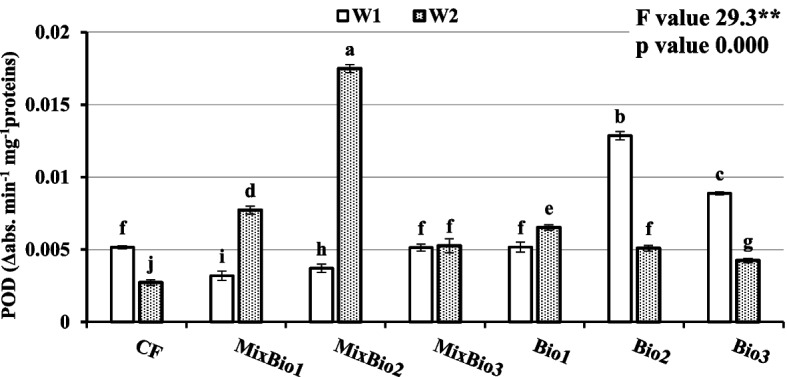
The activity of peroxidase enzyme (POD) of *Moringa oleifera* plants as affected by two water levels control (W_1_) and water stress (W_2_), and different fertilizers. Different litters are significantly different at *p* < 0.05, (mean ± SE; *n* = 3). Interaction F value and *p* value at *P* < 0.05 level. ** high significant, * significant, NS non-significant

From the data of CAT activities represented in Fig. 3 there is highly significant difference between fertilizer treatments. Also, highly significant interaction was recorded between water levels and fertilizers. Bio_3_ biofertilizer induced the highest CAT activity under the two water treatments W_1_ and W_2_. Biofertilizers MixBio_3_, Bio_1_, Bio_2_ and MixBio_1_ induced CAT enzyme activity under drought stress treatment as compared to the corresponding treatments under optimal water level W_1_. On the other hand, MixBio_2_ hindered the activity of CAT under waters tress level W_2_ as compared to the corresponding treatment under optimal irrigation (W_1_). Generally, there was no significance change in POD between the two water levels (Fig. 4). While highly significant change was observed among different fertilizers. Many biofertilizer treatments induced the activity of POD under W_1_ or W_2_ levels. Under drought condition W_2_ biofertilizers MixBio_2_, MixBio_1_ and Bio_1_, respectively induced POD activity when compared to chemical fertilizer control and corresponding treatments under W_1_. While Bio_2_ and Bio_3_ induced POD activity under W_1_ treatment as compared to chemical fertilizer control and corresponding treatments under drought stress W_2_.

**Fig. 5:**
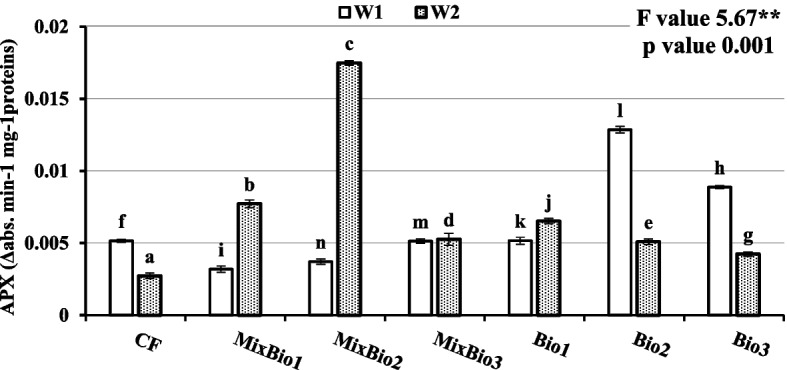
The activity of ascorbate peroxidase enzyme (APX) of *Moringa oleifera* plants as affected by two water levels control (W_1_) and water stress (W_2_), and different fertilizers. Different litters are significantly different at *p* < 0.05, (mean ± SE; *n* = 3). Interaction F value and *p* value at *P* < 0.05 level. ** high significant, * significant, NS non-significant

From the data presented in the (Fig. 5) there was significant interaction between water levels and different fertilizers, generally high significant activity of APX was observed under both water levels. Significant induction in APX activity was observed under different fertilizers, particularly MixBio_2_ under drought stress W_2_. Also, Water stress level W_2_ induced APX under biofertilizers in descending order MixBio_1_ > Bio_1_ > MixBio_3_ > Bio_2_ > Bio_3_ as compared to chemical fertilizer control. While Bio_2_ and Bio_3_ induced the APX under optimal water level W_1_.

### Principal component analysis, hierarchical clustering pattern and correlation analysis

**Fig. 6:**
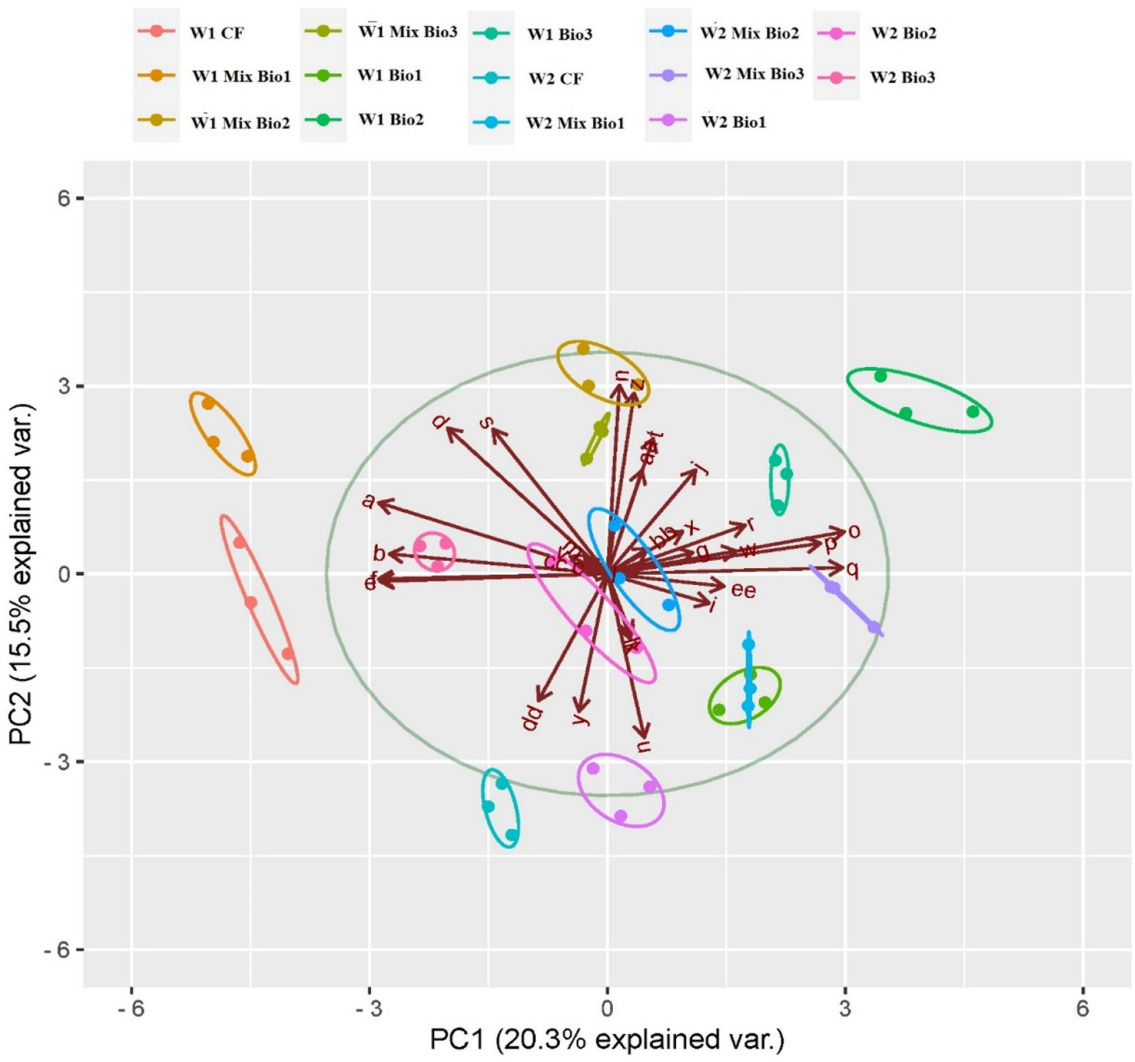
Principal component analysis (PCA) of the studied parameters *Moringa oleifera* plants as affected by two water levels control (W_1_) and water stress (W_2_), and different fertilizers. Abbreviations:** a-** Fwt; **b-** Dwt; **c-** branch number.; **d**- plant height; **e-** dry leaves; **f**- dry leaves yield; **g-** ch.a; **h-** ch.b; **i-** carotenoids; **j**- K^+^ available; **k**- Ca^+2^ available; **l**- P available; **m**- N available; **n-** proteins; **o-** total antioxidants; **p-** reducing power; **q-** flavonoids; **r-** phenolics; **s-** proline; **t**- vitamin C.; **u**- PAL; **v-** PPO; **w-** POD; **x-** CAT; **y-** APX;** z**- OM; **aa**- CEC; **bb-** PH; **cc-** K^**+**^ content in plant; **dd-** N content in plant; **ee**- P content in plant

The principal component analysis (PCA) was computed on the experimental dataset including 32 physiological variables and 14 treatments to enhance the discrimination power to group the measured traits based on relationships among treatments with chemical fertilizer or biofertilizers amendment under drought stress conditions. Since the first two PCs showed the highest percentage of variance, they were used to create a PCA-based biplot. (Fig. 6). Subjection of all the original data of measured traits to PCA gives clear details for all possible correlations (positive & negative) among all assessed traits. The level of trend-similarity is shown by the distances between the qualities on both axes. The biplot of PCA showed contrariness between antioxidants (total antioxidants, flavonoids, phenolics, vit. C., carotenoids, POD, CAT, reducing power) in addition to CEC, pH, phosphorous content in plant (the right-hand half of the biplot Fig. 6) and growth indicators (fresh weight, dry weight, plant height, leaves dry weight, leaves dry yield) in addition to available nitrogen, nitrogen content in plant, and proline (the left-hand half). The first two principal components (PCs) explained a total of 35.8% of the cumulative variance percent (20.3% for the first Axis PC1 and 15.5% for the second axis PC2). The right-hand half of (Fig. 6) was greatly affected by the treatments; W_1_Bio_1_, W_1_Bio_2_, W_1_Bio_3_, W_2_MixBio_2_, and W_2_MixBio_3_.

Meanwhile, the left-hand half was greatly affected with W_1_CF, W_1_MixBio_1_, and W_2_Bio_3_.

**Fig. 7:**
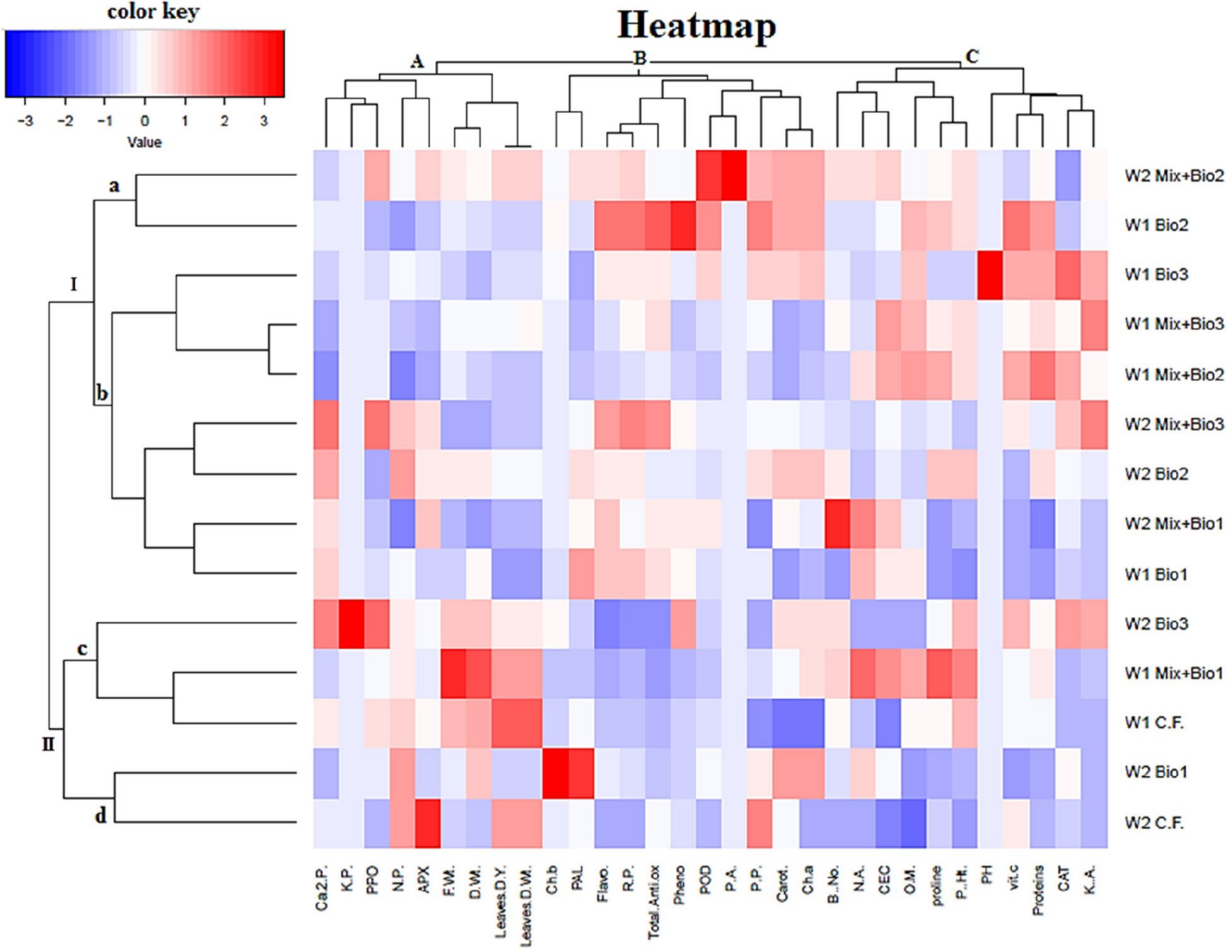
Heatmap showing the saturation of colors indicating effects of the two water levels control (W_1_) and water stress (W_2_), and different fertilizers on the studied parameters in shoot of *Moringa oleifera* plants. Abbreviations: Ca^+2^p calcium content in plant; K.P. Potassium content in plant; PPO polyphenol oxidase, N.P. nitrogen content in plant; F.Wt. fresh weight; D.Wt. dry weight; Leaves D.Y. leaves dry yield; Leaves D.Wt. leaves dry weight; Ch.b chlorophyll b; PAL; Phenylalanine ammonia lyase; flavo flavonoids; R.P. reducing power; Total anti ox total antioxidants; pheno phenolics; POD peroxidase; P.A. phosphorous available; P.P. phosphorous content in plant; Carot. carotenoids; Ch.a chlorophyll a; B.No. Branch numbers; N.A. Nitrogen available; CEC cation-exchange capacity; O.M. organic matter; P.Ht. plant height; Vit.C. Vitamine C.; CAT catalase; K.A. potassium Available

The two-sided dendrogram obtained from the cluster analysis showed that all investigated treatments and measured traits were grouped into different subclusters (Fig. 7). The visual plot of heatmap is used to find complex associations among multiple parameters under different treatments. It is very useful to add hierarchical clustering to heat map, as an attempt to arrange items in a hierarchy according to similarity among them. Hierarchical clustering analysis and heatmap identified the significant differences among treatments on left side and parameters on top (Fig. 7). Biofertilizers and chemical fertilizer applications changed the response of all studied growth, physiological and yield attributes under the two water levels (Fig. 7). It was observed that growth attributes clustered with nutrient content in plant (N, K, Ca^+2^) and enzymes (APX, PPO) as observed in the heatmap (Cluster A). While photosynthetic pigments, PAL enzyme in addition to antioxidants (phenolics, flavonoids, total antioxidants, reducing power, POD) and available phosphorous in soil & phosphorous content in plant were clustered together (Cluster B).

**Fig. 8:**
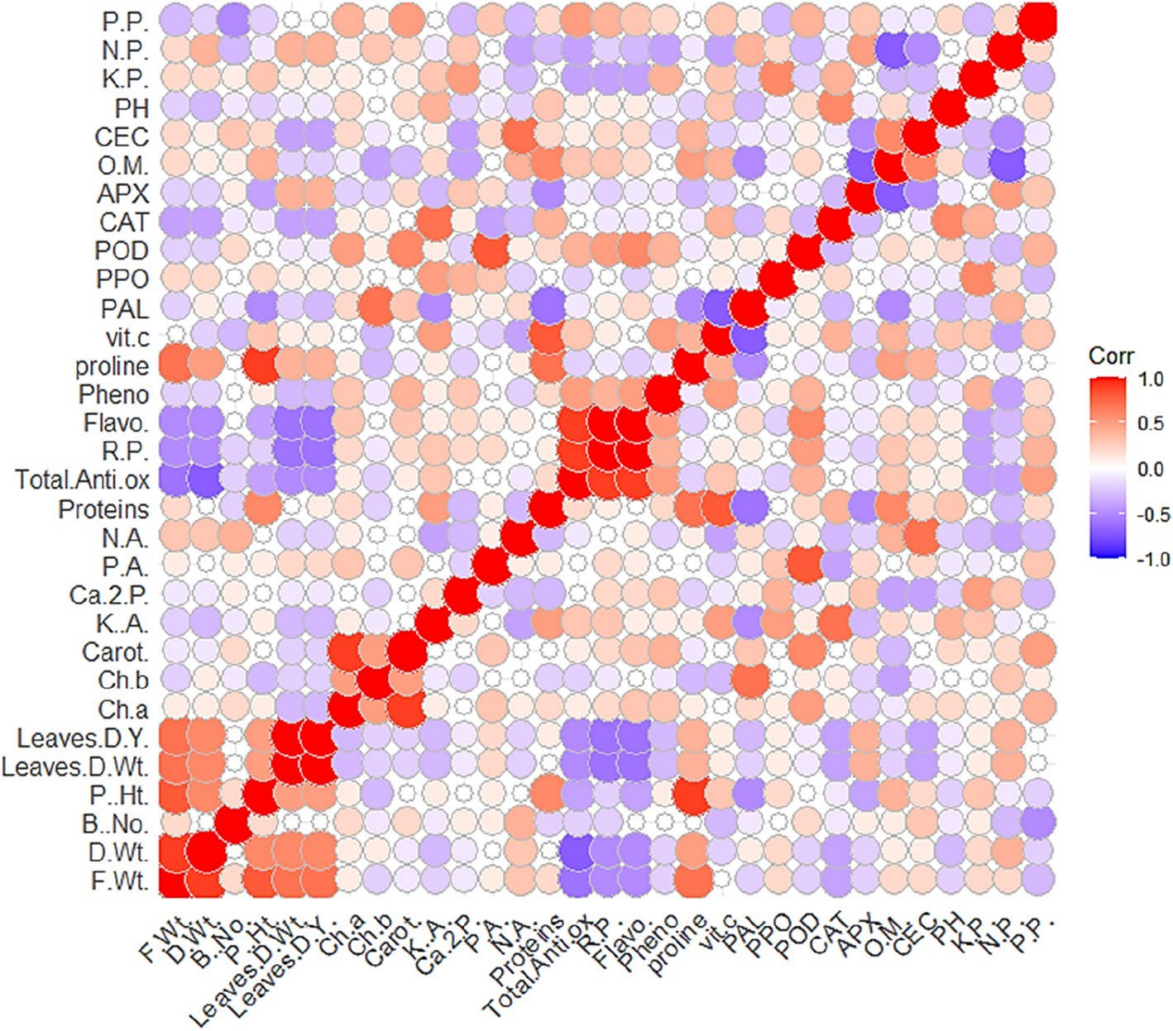
Correlation matrix of the 28 measured traits of the studied parameters in shoot of *Moringa oleifera* plants as affected by two water levels control (W_1_) and water stress (W_2_), and different fertilizers. The increasing color intensities illustrate a higher correlation coefficient. Abbreviations: Ca^+2^p calcium content in plant; K.P. Potassium content in plant; PPO polyphenol oxidase, N.P. nitrogen content in plant; F.Wt. fresh weight; D.Wt. dry weight; Leaves D.Y. leaves dry yield; Leaves D.Wt. leaves dry weight; Ch.b chlorophyll b; PAL; Phenylalanine ammonia lyase; flavo flavonoids; R.P. reducing power; Total anti ox total antioxidants; pheno phenolics; POD peroxidase; P.A. phosphorous available; P.P. phosphorous content in plant; Carot. carotenoids; Ch.a chlorophyll a; B.No. Branch numbers; N.A. Nitrogen available; CEC cation-exchange capacity; O.M. organic matter; P.Ht. plant height; Vit.C. Vitamine C.; CAT catalase; K.A. potassium Available

Visual plot of correlation analysis is used to find positive and negative correlations visually among multiple parameters under different treatments (Fig. 8). Strong negative correlations were observed between flavonoids, reducing power and total antioxidants from one side and growth attributes (fresh weight, dry weight, leaves dry weight, leaves dry yield). Strong positive correlation could be noticed between these parameters (leaves dry weight, leaves dry yield, proline) from one side and (fresh weight, dry weight). Another positive correlation observed between reducing power and flavonoids from one side and total antioxidants from the other side.

## Discussion

Water scarcity in soil causes a variety of physiological and biochemical changes that eventually limit crop productivity and plant growth [72]. Applying plant growth-promoting rhizobacteria (PGPR) in the form of biofertilizers is an efficient way to maintain plant growth and increase productivity under such stressful circumstances. The current study clarifies the function of PGPR-based biofertilizers in reducing the effects of drought stress on *Moringa oleifera* plants and enhancing their ability to recover under limited irrigation.

### Growth performance and recovery after the drought

The application of the biofertilizer Microbine, either alone or in conjunction with compost and rock phosphate, along with the individual application of Potassiumag, greatly enhanced moringa growth under drought stress, according to a comparative analysis of growth parameters during the second cutting. This progress was evident in increased leaf dry weight, improved shoot fresh and dry weights, increased number of branches, and enhanced plant height when compared to chemically fertilized plants (Tables 5 and 6). These findings are in line with earlier studies showing that PGPR application mediates osmotic stress amelioration and improves plant growth under drought conditions [40, 73–77]. Similarly, Al-Fraihat et al. [78] showed that, in comparison to non-inoculated plants, marjoram plants treated with biofertilizer showed the highest values of plant height and branch number.

### Soil physicochemical properties

The soil chemical environment was considerably modified by the application of bio-organic amendments, which resulted in a more fertile rhizosphere for *M. oleifera*. The observed increase in organic Matter (OM) content, particularly in the MixBio_2_ plots, is attributed to the direct input of stabilized carbon by the compost and stimulation of the indigenous microbial biomass. This OM enrichment acts as a nutrient bank and increases the soil’s buffering ability [79]

The significant decrease in soil pH (from 8.05 to approximately 7.36) in the plots treated with bio-organic fertilizer is an important result. This acidification is likely related to the metabolic activities of PGPR (Phosphate-solubilizing and N_2_-fixing bacteria), which release organic acids (e.g., gluconic and citric acids) and protons during the mineralization of organic matter and solubilization of rock phosphate [80]. This effect of localized pH reduction is critical in enhancing the bioavailability of Phosphorus (Ava-P) and other micronutrients which are otherwise fixed in alkaline calcareous soils [81, 82].

The increase in Total Nitrogen (TN) and Available Nitrogen (Ava-N) indicates the efficiency of N_2_-fixing bacteria in the bio-fertilizers and the slow-release nitrogen from compost decomposition [83] for macronutrient dynamics. In addition, the increase in Total and Available Potassium (TK & Ava-K), particularly in the Potassiumag treated soil, highlights the significance of K-solubilizing bacteria in transforming insoluble potassium minerals into plant-available forms via acidolysis and chelation mechanisms [84].

Another long-term advantage of these green technologies is the improvement of the Cation Exchange Capacity (CEC) of MixBio-treated soils. Higher CEC levels enhance the soil’s ability to hold essential cations (Ca^2+^, Mg^2+^, K^+^), thus preventing nutrient leaching and providing a continuous supply of nutrients during the long growth phases of *M. oleifera* growth [85].

### Macronutrient uptake (N, P, K and Ca) under drought conditions

The observed enhancement in macronutrient contents (N, P, K and Ca) was probably attributed to the improvement in soil physicochemical properties by the application of biofertilizer. In particular, the decrease in soil pH and the increase in CEC (Table 1) facilitated the mobilization of the fixed nutrients. This change in chemical properties, probably because of the secretion of organic acids by the introduced PGPR, converted the insoluble minerals into forms available for plant uptake, circumventing the normal process of nutrients becoming fixed in alkaline soils during drought [86].

In comparison to chemical fertilizers, the current study shows that biofertilizer treatments are a promising agricultural strategy for enhancing soil chemical characteristics and nutrient availability. Under conditions of water scarcity, Microbine and potassiumag directly increased nitrogen content, which improved nitrogen uptake. On the other hand, applying Microbine with or without compost and rock phosphate raised the K⁺ content, which is known to be essential for controlling plant growth. Additionally, under drought conditions, Phosphorine application—either by itself or in combination with compost and rock phosphate—increased the Ca content. Potassiumag and Microbine treatments came next. The application of bio-fertilizers helped in maintaining nutrient levels close to those of the control even under drought conditions, preventing the drastic decline typically observed under stress. Ti et al. [87] found that drought dramatically raised the amount of calcium in maize grains while lowering the concentrations of P and K. These changes were attributed to changes in the availability and transport of minerals under low soil moisture. On the other hand, Oktem [88] proposed that poor mineral mobilization brought on by water scarcity is the main cause of decreased mineral uptake during drought. The present results align with previous studies [40, 78, 89]. While Ghoneim and EL-Araby [90] found increased N, P, Ca, and K concentrations in Jew's mallow leaves after biofertilizer inoculation, Zayed [91] reported increased Mg, P, K, Mn, Fe, and Cu contents in *Moringa oleifera* seeds after soil inoculation with mixed microbial cultures.

Multivariate analysis offered an integrated interpretation of the intricate reactions of moringa plants to drought stress and biofertilizer application in addition to univariate comparisons. This allowed principal component analysis to investigate the growth parameters, nutritional status, photosynthetic pigments, osmolytes, and antioxidants in one go. This also enabled the understanding of the relationship of these parameters to different levels of fertilization treatments.

The first principal component was dominantly linked with variables of growth (height of plants, number of branches, fresh and dry weight of shoots, and leaf dry weight) and the composition of macronutrients (N, P, K, and Ca). This classification again supports the concept that superior growth potential under biofertilizers was inextricably linked with superior utilization of nutrients under water-stressed conditions.

The second principal component was mainly influenced by photosynthetic pigments, osmotic adjustment markers, and parameters associated with antioxidants such as chlorophyll a, chlorophyll b, carotenoids, proline, total antioxidants, and reducing power. This shows that the capacity for photosynthesis and osmotic adjustment in drought-stressed plants was associated with the induction of the antioxidant system. This integration confirms that the physiological resilience of drought-stressed plants is a coordinated response where photosynthetic stability and osmotic adjustment are supported by the induction of a robust antioxidant system.

Further, hierarchical cluster analysis showed the significance of the results mentioned above by grouping bio-fertilizers, particularly the combination of Microbine, Potassiumag, and Phosphorine, together with compost and rock phosphate, in clusters different from those of chemical fertilizer. These clusters were differentiated based on higher growth, higher nutrient content, enhanced stability of photosynthetic pigments, and higher antioxidant activity, thereby proving the cumulative effect of PGPR bio-fertilizers in reducing drought stress.

### Nitrogen uptake and photosynthetic pigments

Following the application of microbine, potassium, and phosphorus, the amounts of photosynthetic pigments in moringa leaves, such as carotenoids, chlorophyll a, and chlorophyll b, significantly increased, improved plant growth and productivity were a direct result of these higher pigment levels. Nitrogen content data (Table 7) demonstrated the capacity of these biofertilizers to fix atmospheric nitrogen and supply it to moringa plants, as PGPR application-maintained nitrogen levels similar to those attained by chemical fertilization. The observed increases in chlorophyll a, chlorophyll b, and carotenoid contents were probably caused by this increased nitrogen availability.

Microbine, Potassiumag, and Phosphorine consist of a consortium of nitrogen-fixing, potassium, and phosphate-solubilizing bacteria that can produce organic acids, growth-promoting phytohormones like indole-3-acetic acid (IAA), and advantageous enzymes [30]. These traits have a direct impact on the physiological and biochemical functions of moringa plants, leading to enhanced stabilization and chlorophyll biosynthesis under various water regimes. Previous reports have documented similar improvements in photosynthetic pigments after PGPR application [92, 93].

### Soluble protein accumulation

The soluble protein accumulation varied greatly among fertilizer treatments, with the highest accumulation in plants receiving Microbine alone or in combination with rock phosphate and compost, and then after Potassiumag application. This could be attributed to the improvement in the availability of nutrients, such as nitrogen, brought about by the biofertilizers. These results are in agreement with the previous studies [[92, 93]. On the other hand, there are studies showing that unfavorable growth conditions, such as drought stress, could also lead to the production of proteins [94, 95].

### Oxidative stress and antioxidant defense system

Water stress triggers oxidative stress in plants because of overproduction of ROS, which can cause cellular damage if it is not mitigated by a strong antioxidant defense mechanism [90] [96]. This defense mechanism is made up of enzymatic antioxidants like catalase (CAT), peroxidase (POD), and ascorbate peroxidase (APX), and non-enzymatic compounds such as ascorbic acid, proline, phenolics, flavonoids, and carotenoids [97].

The significant increase in the activity of antioxidant enzymes, particularly CAT, POD, and APX in bio-fertilized *M. oleifera* plants under drought stress (W2) suggests a strong adaptive strategy to minimize oxidative damage. These enzymes serve as a cooperative defense system to scavenge ROS such as hydrogen peroxide (H_2_O_2_) to protect cellular membranes against lipid peroxidation. The higher efficacy of treatments such as Bio_3_ for CAT induction and MixBio_2_ for APX induction indicates that PGPR strains (*Bacillus and Azotobacter*) behave as biological elicitors. These microbes induce “induced systemic tolerance” (IST) by modulating the plant hormonal balance and promoting the expression of antioxidant defense genes [98, 99]. In particular, the induction of APX, which has a higher affinity for H_2_O_2_ than CAT, suggests an efficient fine-tuning of the ascorbate–glutathione cycle under prolonged stress, to protect the photosynthetic apparatus [100].

The presence of higher levels of soluble proteins in both Microbine and Potassiumag indicates their capability to achieve efficient nitrogen utilization (NUE). The reason behind this conclusion is the ability of plants to have sustained their nitrogen absorption at high levels (Table 7) despite being subjected to water deficiency, thus allowing them to support the metabolic activities needed in protein biosynthesis along with the formation of new antioxidant enzymes (CAT, POD, and APX) [96, 97].

Bio-fertilizers containing beneficial bacterial cultures were observed to increase the enzymatic as well as non-enzymatic antioxidants in moringa plants, thus reducing the effects of water deficit conditions. The use of Phosphorine, Microbine, and Potassiumag increased the antioxidant defense mechanism in plants, thus aiding in maintaining cellular equilibrium under drought conditions. Although the ability of plants to resist oxidative stress has been widely investigated under adverse conditions of the environment [101, 102], very little is known about the dynamics of antioxidants during the reproductive phase of plants.

### Non-enzymatic antioxidants and reducing power

In the current research, the non-enzymatic antioxidants, such as proline, ascorbate, and carotenoids, in addition to the enzymatic antioxidants, such as POD, APX, and CAT, were investigated. In addition, the total antioxidant capacity and the reducing power of the leaves of the moringa plant grown under drought stress were also determined.

Multivariate correlation patterns revealed strong positive associations between nutrient elements, particularly nitrogen and potassium, and antioxidant enzymes such as CAT, POD, and APX, as well as non-enzymatic antioxidants including phenolics, flavonoids, carotenoids, and proline. This relationship suggests that improved nutritional status under biofertilizer application supported the metabolic energy required for antioxidant synthesis and enzyme activation, thereby enhancing oxidative stress tolerance under drought conditions.

The co-loading of PAL, PPO, phenolics, and flavonoids in multivariate space confirms that the phenylpropanoid pathway operated as an integrated defensive module rather than as independent biochemical reactions. This coordinated induction contributes to efficient ROS detoxification and cellular protection during prolonged water deficit.

In general, reducing power increased under most biofertilizer treatments, although Potassiumag applied individually did not induce a similar response. Conversely, Phosphorine and Potassiumag combined with rock phosphate and compost enhanced total antioxidant content under drought conditions compared with chemical fertilization.

Proline serves as a vital physiological indicator of stress tolerance, contributing to osmotic adjustment, free radical scavenging, and stabilization of subcellular structures under water stress [103]. While moringa plants generally did not show increased proline accumulation under drought alone, Microbine and Potassiumag applications, either individually or combined with compost and rock phosphate, significantly enhanced proline content relative to chemical fertilizer.

Previous studies on tomato plants reported drought-induced increases in ascorbate content [104, 105], and salinity stress was shown to enhance de novo ascorbate synthesis [106, 107]. In the present study, Potassiumag was the only biofertilizer that increased ascorbate content under both normal and water-deficient conditions compared with chemical fertilizer. Other biofertilizers did not show similar effects, possibly due to the complex regulation of ascorbate metabolism. Previous reports on ascorbate responses under stress have been inconsistent [108, 109] indicating that severe stress may disrupt ascorbate biosynthesis or recycling.

### Phenylpropanoid metabolism and enzymatic antioxidants

Phenolic compounds, including flavonoids, anthocyanins, tannins, and related metabolites, constitute a diverse class of antioxidants synthesized through the phenylpropanoid pathway [110, 111]. These compounds play a critical role in plant defense against abiotic stress. Phenylalanine ammonia lyase (PAL), a key enzyme in this pathway, is known to be strongly induced by stress conditions [112, 113], leading to enhanced phenolic accumulation.

Regarding the secondary metabolism, the significant increase in PAL activity, particularly in the Bio_1_ treated plants, indicates activation of the phenylpropanoid pathway. PAL is a key regulatory enzyme in the biosynthesis of phenolic compounds and flavonoids that act as non-enzymatic antioxidants, quenching ROS and screening against environmental stressors. The induction of PPO also indicates an increased potential for the oxidation of phenols to quinones, thus providing a second layer of biochemical defense [114]. The synergy observed in MixBio treatments could be attributed to the improved nutrient availability in terms of Nitrogen and micro-nutrients supplied by compost and rock phosphate which serve as the precursors and co-factors for the synthesis of these proteinaceous enzymes. This metabolic “priming” by bio-organic amendments allows the plant to maintain physiological homeostasis even at limiting soil water potential [115].

In the present study, biofertilizer application under drought conditions significantly affected flavonoid and phenolic contents in moringa leaves. The highest flavonoid concentrations were recorded in Potassiumag, Phosphorine, and Microbine treatments combined with rock phosphate and compost. Phenolic content was highest under Microbine applied alone, followed by Phosphorine, Potassiumag, and Microbine combined with rock phosphate and compost. These trends were consistent with PAL activity, which showed marked induction under biofertilizer treatments during drought stress.

Polyphenol oxidase (PPO), another key enzyme involved in phenolic metabolism, generally responds actively to plant stress [116]. PPO and related enzymes participate in ROS detoxification either directly or through reactions mediated by peroxidases [117]. In this study, PPO activity increased under drought conditions, particularly following Potassiumag application alone or combined with rock phosphate and compost, followed by Microbine combined with rock phosphate and compost, and individual Phosphorine application. These findings are consistent with previous reports documenting increased PPO, POD, and PAL activities under biotic and abiotic stress conditions [118].

The joint induction of the activity of PAL together with the increased content of phenolics and flavonoids (Figs. 1 and 2) provides evidence of phenylpropanoid pathway induction. From the point of view of the multivariate correlation patterns, the strong positive correlation between the nutrient content and these metabolites suggests that the process of acquiring nutrients by the PGPR led to the accumulation of carbon skeletons and energy sources required for secondary metabolism. This integration, where PPO and PAL work in association, underscores a systemic adaptive response rather than isolated biochemical events [112, 118].

### Enzymatic antioxidant responses

Induction of the antioxidant enzyme system represents a critical strategy for detoxifying ROS generated under abiotic stress [119]. CAT, APX, and POD are major enzymes involved in ROS scavenging [120]. Hydrogen peroxide (H₂O₂), a toxic ROS, must be rapidly converted to water and oxygen by CAT or peroxidases to prevent cellular damage [121, 122].

In the present study, almost all biofertilizer treatments enhanced CAT and POD activities under water deficit conditions compared with chemical fertilizer. Moreover, under drought stress, moringa plants exhibited a marked increase in APX activity following Microbine and Phosphorine applications combined with rock phosphate and compost. This response highlights the crucial role of APX in H₂O₂ scavenging and efficient oxidative stress management, ultimately contributing to improved drought tolerance.

### Integrative interpretation of drought tolerance mechanisms

Overall, multivariate analysis strengthened the interpretation derived from univariate results by demonstrating that drought tolerance in moringa plants was achieved through coordinated physiological, biochemical, and metabolic adjustments. Growth recovery, nutrient uptake, photosynthetic stability, osmotic regulation, and antioxidant defense were not independent responses but formed an interconnected adaptive network promoted by biofertilizer application. These findings emphasize that the beneficial effects of PGPR-based biofertilizers under drought conditions are systemic rather than trait-specific, providing a comprehensive mechanistic explanation for the enhanced performance of moringa plants compared with chemical fertilization alone.

**Fig. 9:**
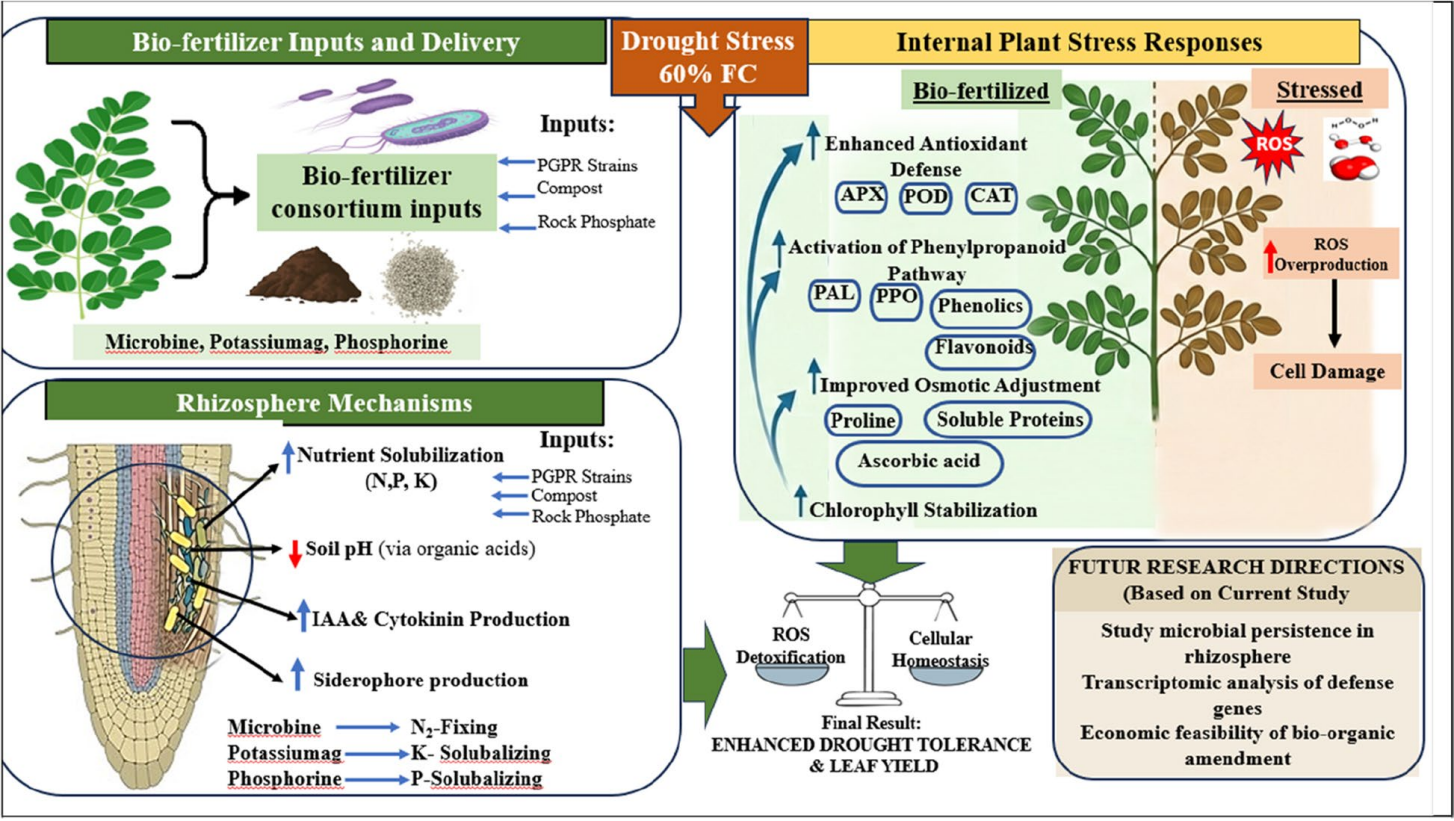
A conceptual mechanistic model illustrating the synergistic effects of PGPR-based biofertilizers on Moringa oleifera under drought stress. The model highlights how bio-organic amendments improve soil health (↑OM, ↓pH), leading to enhanced nutrient uptake and the subsequent activation of antioxidant defense systems (enzymatic and non-enzymatic) and osmotic adjustment, collectively fostering physiological resilience and growth recovery

Figure 9 summarizes and illustrates the integrated mechanistic pathways of PGPR-based biofertilizer mitigation of drought stress in *M. oleifera*, from soil nutrient mobilization to the induction of enzymatic and non-enzymatic antioxidant defense systems.

#### Study limitations and perspectives

Although this study provides insights into the biological contribution of biofertilizer in improving Moringa’s resistance against drought stress, it should be mentioned that such responses are associated with a particular type of sandy loam soil and a period of two harvests. Further research could include an investigation of the long-term effect of microbes in the rhizosphere and molecular transcriptomic analysis of defense genes to gain more knowledge about signaling pathways between the plant and microbes. Nevertheless, current results indicate strongly the use of PGPR as an effectiveness tool for sustainable production in arid regions.

## Conclusion

The current study provides evidence on the potential application of bio-organic technology- incorporating phosphate solubilizers, nitrogen fixers, and potassium solubilizers- as effective sustainable strategy for improving *Moringa oleifera* performance during optimal (100% FC) and deficient (60% FC) watering regimes. The results demonstrate that the combined application of Microbine with compost and rock phosphate can be considered as a potent sustainable alternative to chemical fertilizers. Indeed, these factors considerably increased biomass production, dry leaf yield, and functional metabolic synthesis such as proteins, proline, and ascorbic acid under optimal soil moisture. However, under drought conditions, individual application of Potassiumag and Phosphorine proved to be sufficient soil amendment for sustaining physiological equilibrium. Such resilience can be attributed to a systemic adaptive process, including efficient nutrient metabolism (primarily K⁺ osmoregulation) and increased enzymatic and non-enzymatic antioxidants defense systems.

In addition to improving crop yield, these bio-organic treatments enhanced the nutritional value and antioxidant activity of the *M. oleifera* leaves, providing an effective approach to manufacture high-value dietary supplements. Overall, these findings form a sound scientific foundation for moving from chemical fertilizers to sustainable practices in dry regions amid global warming threats.

Limitations and Future Perspectives: It must be emphasized that although these results are encouraging, they apply only to the sandy loam soil type and two harvests within the scope of the present investigation. Further research needs to explore the survival and colonization patterns of the inoculated PGPR bacteria over the long term. Moreover, elucidation of the underlying molecular signaling events and a comprehensive economic feasibility analysis will be critical to scaling up the utilization of these green bio-organic practices by local agricultural sectors.

## Data Availability

All data generated or analyzed during this study are included in this published article and its supplementary information file is not publicly available due to its proprietary nature. Supporting data cannot be made openly available but are available from the corresponding author on reasonable request.
